# Epidemiological Study of Mammary Tumors in Female Dogs Diagnosed during the Period 2002-2012: A Growing Animal Health Problem

**DOI:** 10.1371/journal.pone.0127381

**Published:** 2015-05-18

**Authors:** Yaritza Salas, Adelys Márquez, Daniel Diaz, Laura Romero

**Affiliations:** 1 Doctoral Program in Production Science and Animal Health, Facultad de Medicina Veterinaria y Zootecnia (FMVZ), Universidad Nacional Autónoma de México (UNAM), Mexico City, Mexico; 2 Pathology Department, Universidad Centroccidental “Lisandro Alvarado” (UCLA), Barquisimeto, Lara, Venezuela; 3 Physiology Department, UCLA, Barquisimeto, Lara, Venezuela; 4 Biology Cellular and Physiology Department, Instituto de Investigaciones Biomédicas, UNAM, Mexico City, Mexico; 5 Pathology Department, FMVZ, UNAM, Mexico City, Mexico; University of Tennessee Health Science Center, UNITED STATES

## Abstract

Epidemiological studies enable us to analyze disease behavior, define risk factors and establish fundamental prognostic criteria, with the purpose of studying different types of diseases. The aim of this study was to determine the epidemiological characteristics of canine mammary tumors diagnosed during the period 2002-2012. The study was based on a retrospective study consisting of 1,917 biopsies of intact dogs that presented mammary gland lesions. Biopsies were sent to the Department of Pathology FMVZ-UNAM diagnostic service. The annual incidence of mammary tumors was 16.8%: 47.7% (benign) and 47.5% (malignant). The highest number of cases was epithelial, followed by mixed tumors. The most commonly diagnosed tumors were tubular adenoma, papillary adenoma, tubular carcinoma, papillary carcinoma, solid carcinoma, complex carcinoma and carcinosarcoma. Pure breeds accounted for 80% of submissions, and the Poodle, Cocker Spaniel and German Shepherd were consistently affected. Adult female dogs (9 to 12 years old) were most frequently involved, followed by 5- to 8-year-old females. Some association between breeds with histological types of malignant tumors was observed, but no association was found between breeds and BN. Mammary tumors in intact dogs had a high incidence. Benign and malignant tumors had similar frequencies, with an increase in malignant tumors in the past four years of the study. Epithelial tumors were more common, and the most affected were old adult females, purebreds and small-sized dogs. Mammary tumors in dogs are an important animal health problem that needs to be solved by improving veterinary oncology services in Mexico.

## Introduction

In Veterinary medicine, mammary tumors represent the most frequently diagnosed neoplasm in intact female dogs, and 50% of these are malignant [1]. A study focusing on the incidence of canine mammary tumors found tumors in approximately 0.05% of females that were spayed before their first heat cycle. This figure increased to 8% or 26% when the animals were spayed after their first or second heat, respectively. However, if the animals were spayed later, the risk of developing malignant tumors (MN) was the same as for an intact bitch [2]. Mammary tumors can vary in size from a few millimeters to over a few centimeters, and at least 50% of the cases present multiple masses mainly located at the caudal glands [3]. As in humans, canine mammary cancer is a heterogeneous group of diseases linked to morphology and biological behavior. Histologically, mammary tumors are classified as malignant epithelial tumors, special types of epithelial tumors (Squamous cell carcinoma, Adenosquamous carcinoma, Mucinous carcinoma, Lipid-rich (secretory) carcinoma, Spindle cell carcinomas and inflammatory carcinoma), malignant mesenchymal tumors, carcinosarcoma and benign tumors (BN) [4].

Mammary gland tumors share common features between dogs and humans. For this reason, they are excellent models for human breast cancer studies and comparative studies in relation to breast cancer prognosis and treatment [1]. Generally, in canine mammary tumors (CMT), especially in metastatic tumors, genes in charge of DNA repair show genetic instability with unknown causes. Nevertheless, it is believed that aberrant tumor cell division with damaged DNA replication, hypoxia, mutations accumulation and DNA repair genes, epigenetic modifications can contribute to this phenomenon [5]. The continuous cell proliferation caused by mutationally activated proto-oncogenes or tumor suppressor gene inactivation induces the replication of damaged DNA [6]. Additionally, chronic hypoxia or hypoxia and re-oxygenation cycles contribute to genomic instability [7].

In accordance with the Minister of Health, in Mexico City, there are an estimated 1.2 million dogs. However, animal rights organizations declared an estimate between 3 and 5.5 million dogs [8]. Taking into consideration the canine population and the high incidence of mammary tumors in this species, the disease can be considered a major animal health problem, requiring diagnostic and therapeutic alternatives as well as an outstanding infrastructure to address the issue. Given this scenario, it is mandatory that public and private veterinary hospitals diagnosing canine mammary tumors should increase their investment in order to properly manage the increasing number of patients affected by this disease. In this context, the veterinary professional’s preparation, along with scientific breakthroughs, provides adequate support for diagnostic and therapeutic strategies, which are essential to guarantee the dog’s quality of life and health.

In Veterinary medicine, the increase in the incidence of neoplastic disease (including mammary tumors) requires continuous development from veterinary oncology specialists. In this respect, retrospective epidemiological studies represent a helpful approach and an important source of information for analyzing neoplastic disease behavior over time. These studies are also useful for establishing risk factors and prognosticating criteria from clinical and histopathological features. Therefore, these may be translated into relevant scientific information that may be used as a basis for experimental studies. This work aimed to determine the epidemiological behavior of canine mammary tumors diagnosed at the Department of Pathology, Faculty of Veterinary Medicine (FMVZ) of the National Autonomous University of Mexico (UNAM) during the period from January 2002 to December 2012.

## Material and Methods

This retrospective study focused on reviewing 11,544 dog biopsy samples processed at the Department of Pathology, FMVZ-UNAM (January 2002 to December 2012). The study included 1,917 biopsies of mammary lesions from female dogs. Biopsy files were reviewed, and all of the information regarding mammary tumors (histological types: benign or malignant [9].; non-neoplastic lesions: hyperplasia, dysplasia and / or inflammation) was collected. In every case study, the information about age, sex, breed and hormonal status was obtained. None of the dogs were spayed before the third heat. In fact, most of the animals were spayed at the time of the mastectomy. Breeds were classified in accordance to their cross height (i.e., the highest point at the loin, where it crosses two imaginary lines, a vertical line (front legs) and a horizontal line (spine), in the following way: small (<35 cm), medium (35–50 cm) and large (> 50 cm), in accordance with the FCI (Federation Cynologique Internationale [10]) Breeds Nomenclature. It should be mentioned that the same height parameter was used for the classification of mixed-breed dogs. The specific anatomical location of the tumor was not considered because such information was not always available.

### Statistical analyses

The analysis of data was performed using descriptive statistics and an analysis of variance (ANOVA), as well as bivariate and multivariate analyses. Absolute and relative annual frequencies of the cases were obtained to compare the mammary tumor frequency in female dogs. Cumulative frequencies were compared by fitting Gaussian curves and using the extra sum-of-squares F test to determine if the curves were similar. Furthermore, *X* ^2^ analysis was used for the proportions differentiation to determine if the frequencies of benign or MT (same tissue of origin) were similar.

The studied population was divided in accordance to their size: small, medium and large dogs. Diagnosed females were also classified by age using frequency distributions and constructing histograms.

*X* ^2^ (trend analyses) were used to determine the strength of the associations between the presence of benign or malignant neoplasms and the breed, age group or size category.

A two-way ANOVA, followed by a Sidak’s multiple comparison test, was used to evaluate the differences of the mean annual frequency in benign or MT (according to the tissue of origin). The data are presented as the means ± SE or mean and 95% CI.

Lastly, a correspondence analysis (COA) was used to assess the pattern of association among variables exhibited by the female dog breed, size category, tumor morphology and tissue of origin of the mammary lesion. A modification of a previously reported method was used to achieve this objective [11]. This multivariate analysis uses *X* ^2^-calculated distances to determine the association between groups of variables; for example, a particular breed and any morphological type of mammary lesion. The analysis assumes that, for each group of variables, there is no association between categories. Therefore, the null hypothesis was based on equal distances between variables. In such a case, whenever two categories of a defined variable were closer than any other category, the null hypothesis was rejected, and a correspondence or association was defined for these adjacent points. The correspondence analysis established sets of combinations amongst the variables. The first combination (first factor or Dim 1) defined a scale of variation that helped discriminate groups of variables. The second combination (second factor or Dim 2) defined the separation of non-related groups. The sum of Dim 1 and Dim 2 explained the percentage of the total variance shown by the data.

In all of the cases, a value of *p* < 0.05 was considered significant. Data were analyzed using SAS 9.0 (Statistical Analysis System, SAS Institute, USA) and Prism 6.0 (Graph Pad Inc., USA).

## Results

### Frequency of canine mammary tumors during the time of the study

**Fig 1:**
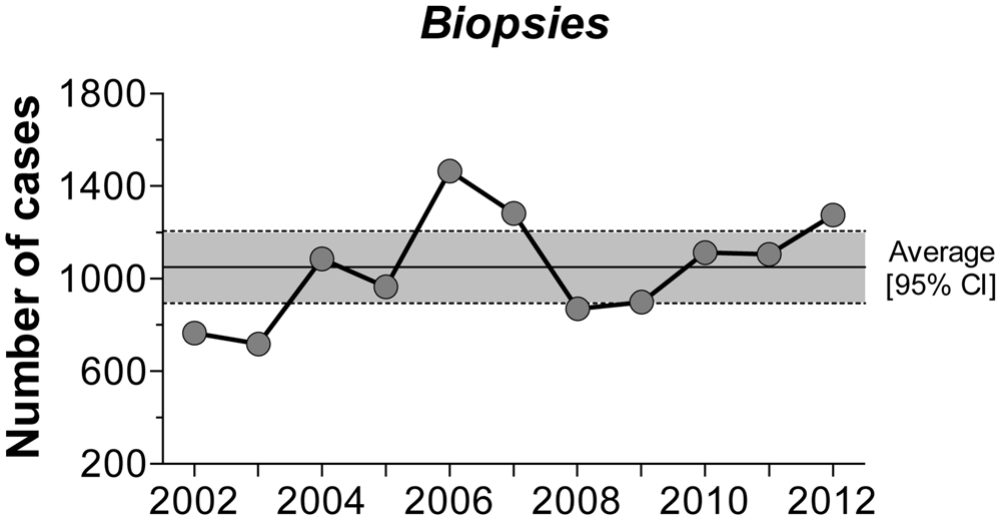
Annual incidence of canine biopsies registered at the Department of Pathology, FMVZ-UNAM (2002–2012).

**Fig 2:**
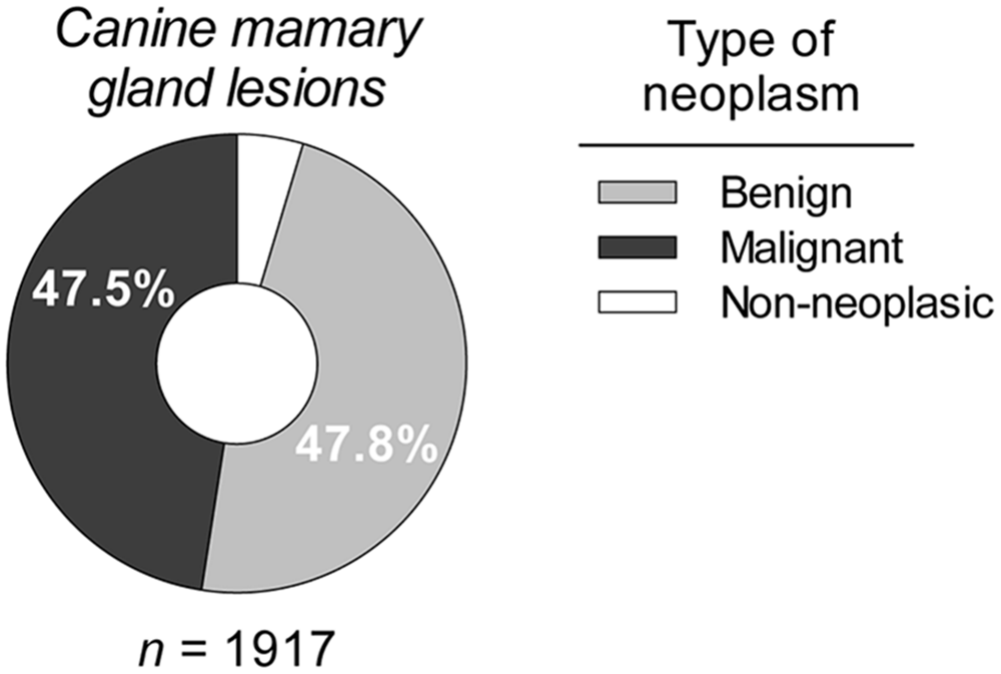
Relative incidence of mammary gland lesions according to biological behavior.

**Fig 3:**
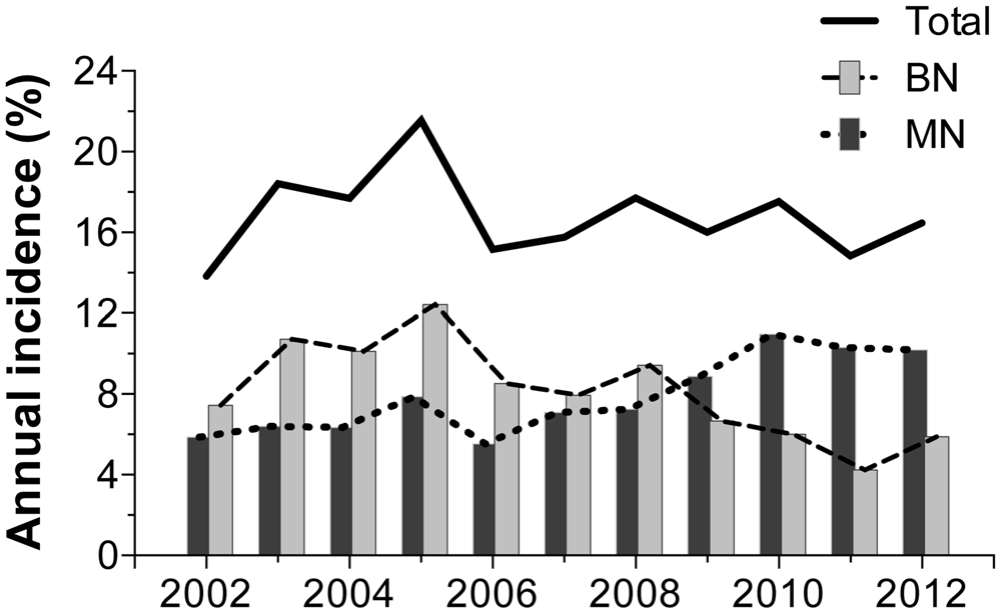
Total annual incidence of canine mammary tumors according to biological behavior.

**Fig 4:**
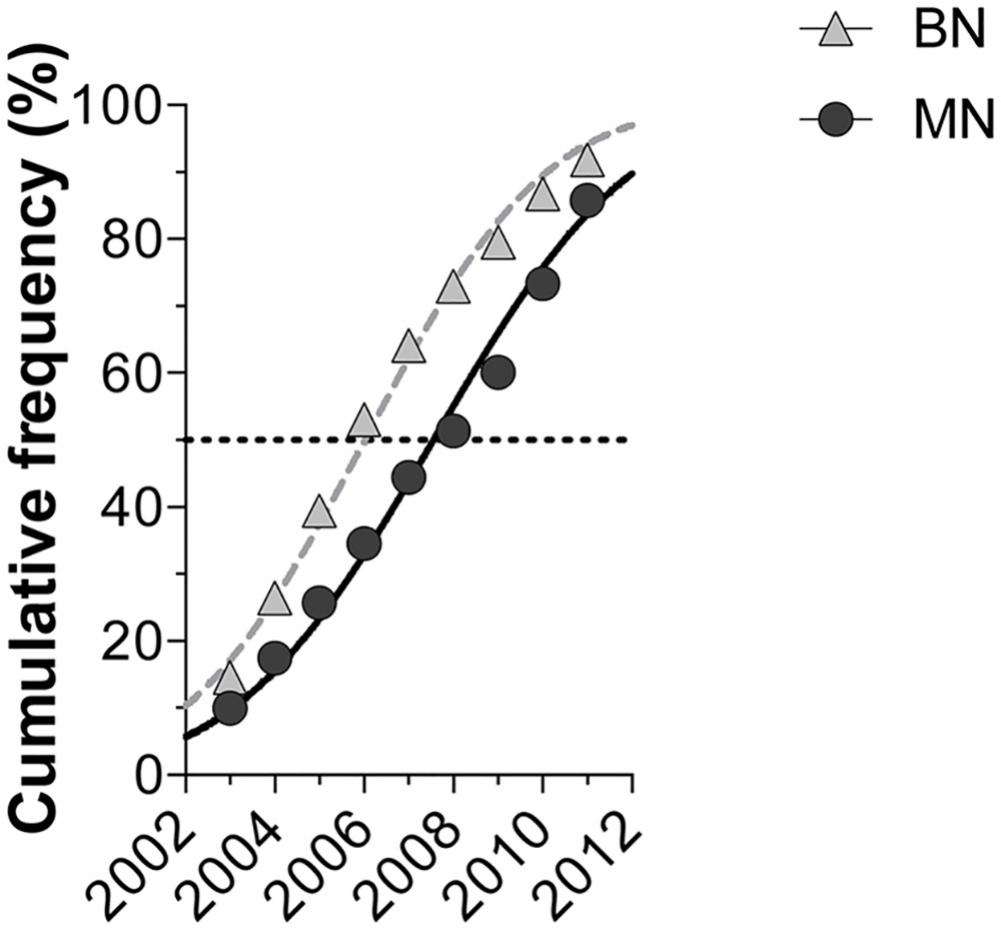
Gaussian-fitted annual cumulative frequency of mammary tumors. BN: Benign tumor; MN: Malignant tumor.

During the 11 years that the study covered, a total of 11,544 tissue biopsies were processed, with an annual average of 1,049 [95% CI: 893.4 to 1206] biopsies (Fig 1). A total of 1,917 processed samples corresponded to mammary gland lesions from intact female dogs (99% of cases of canine mammary tumors—CMT). Of these lesions, 47.8% were BN and 47.5% were MN. Other lesions accounted for the remaining 4.7% and were diagnosed as non-neoplastic disorders, including hyperplasia, dysplasia or inflammation (Fig 2). Fig 3 presents the time series of the total annual frequency of CMTs during the years of study, in accordance with their biological behavior. The average annual frequency of CMTs was 16.8% [95% CI: 15.3 to 18.2]. The mean average frequencies of BN and MN were 8% [95% CI: 6.5 to 7.9] and 7.8% [IC95% CI: 6.5 to 9.1], respectively. Although the frequency of both tumor types was quite similar, the time series showed substantial differences in relation to the biological behavior. The comparison of the adjusted cumulative frequencies depicted in Fig 4 shows that 53% of the cumulative cases of BN were obtained during the period from 2002 to 2006. Conversely, during this period, only 35% of cumulative cases were registered for MN, showing large differences (*p* < 0.001) between the fitted curves and a shift in the accumulation of MN.

### Frequency of occurrence of mammary tumors according to the tissue of origin

**Fig 5:**
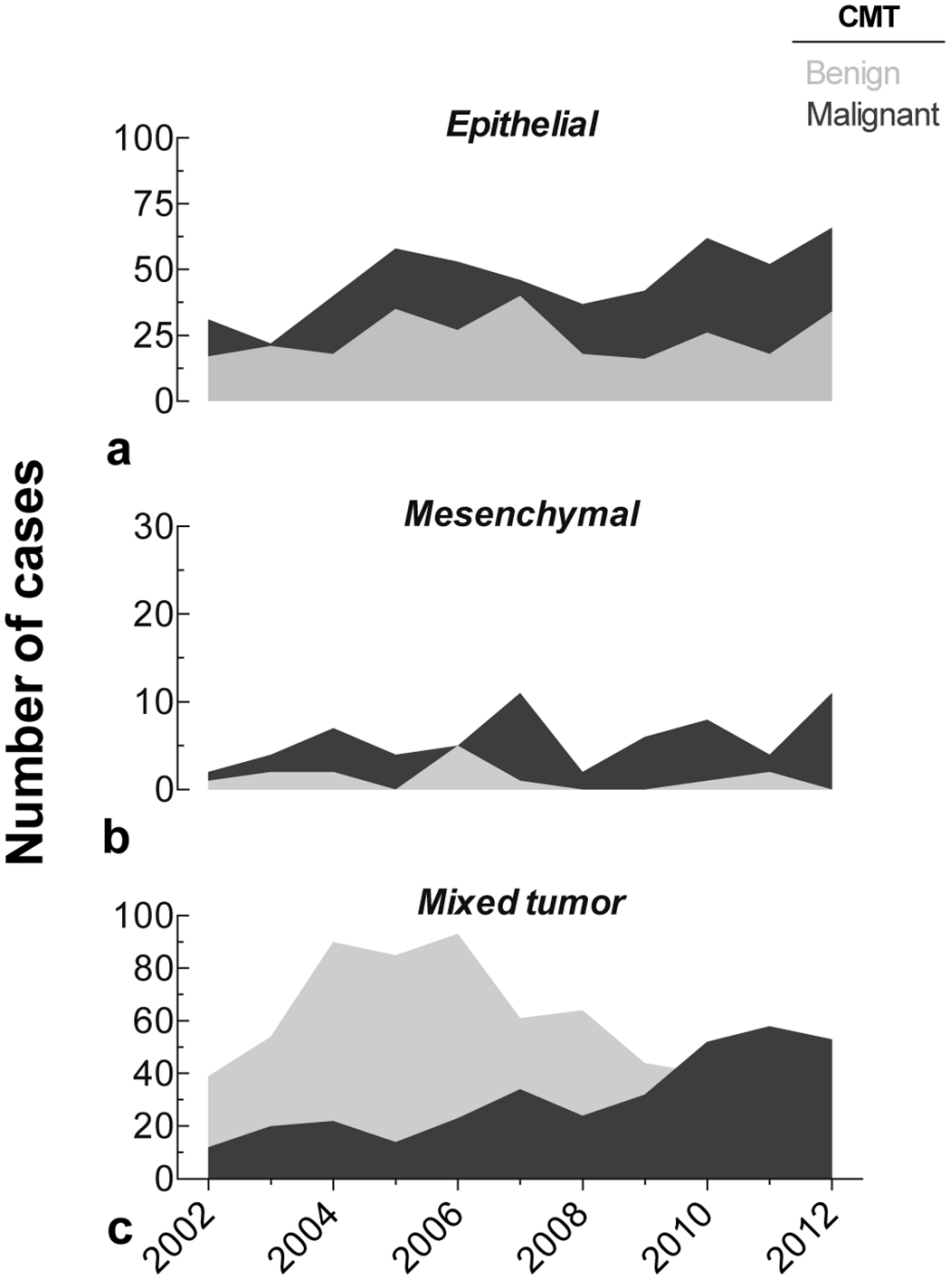
Time series of the number of benign or malignant cases of canine mammary tumors classified according to the tissue of origin: (a) Epithelial, (b) Mesenchymal or (c) Mixed tumor.

**Fig 6:**
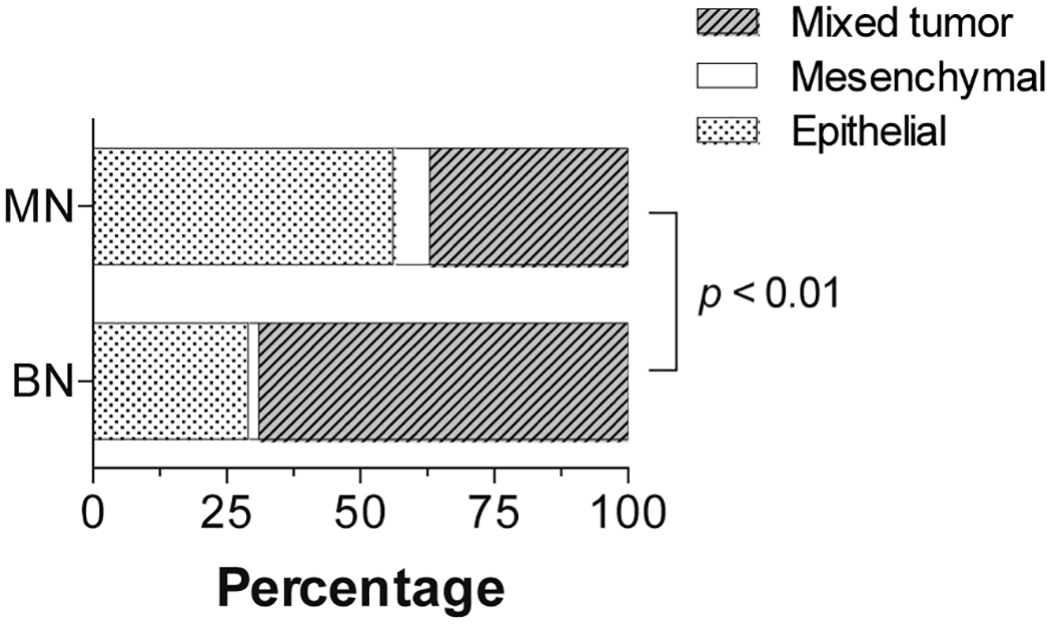
Relative frequency of benign or malignant canine mammary tumors according to the tissue of origin.

During the study period, the comparison of the frequency of occurrence (CMT) with their tissue of origin presented certain changes in the biological behavior of mammary tumors (Fig 5a–5c). MN of epithelial origin showed the highest number of cases, followed by mixed tumors. During 2006 and 2008, a sustained increase was recorded for the occurrence of epithelial and malignant mixed tumors. BN were mostly represented by mixed-origin mammary lesions, and least frequently by epithelial type. Consistently, both MN and BN lesions (mesenchymal origin) were the less frequent. Since 2006, there was a clear decrease in benign mammary lesions (mixed origin), in contrast to the increased number of cases of MN. (Fig 5a–5c). The relative frequency of histological types differed between MN and BN (Fig 6; *p* < 0.01). Epithelial tumors occurred in 69% of the malignant lesions, while benign mixed tumors were more plentiful in comparison to MN (56% and 29%, respectively). The annual frequency of epithelial and mixed tumors was different (*p* < 0.001) between benign and MN (see Table 1). Despite the fact that mesenchymal MN showed an annual prevalence four times higher than BN, no significant difference was found (*p* > 0.05). It is important to mention that highly significant differences were found (*p* <0.001) when comparing the average annual frequencies of the three histological types for each biological behavior.

**Table 1:** Annual frequency of canine mammary tumors according to the tissue of origin (2002–2012).

Tissue of origin	CMT Benign (%)	CMT Malignant (%)	*p* value
Epithelial	30.4 ± 8.9 ^b^	56.8 ± 9.7 ^a^	< 0.001
Mesenchymal	1.5 ± 1.5 ^c^	6.9 ± 2.7 ^c^	> 0.05
Mixed tumor	68.0 ± 8.9 ^a^	36.2 ± 9.1 ^b^	< 0.001

^a,b,c^ For benign or malignant neoplasm, uncommon superscript between tissue origin indicates differences at *p* < 0.001.

### Main morphological diagnosis in canine mammary tumors

**Fig 7:**
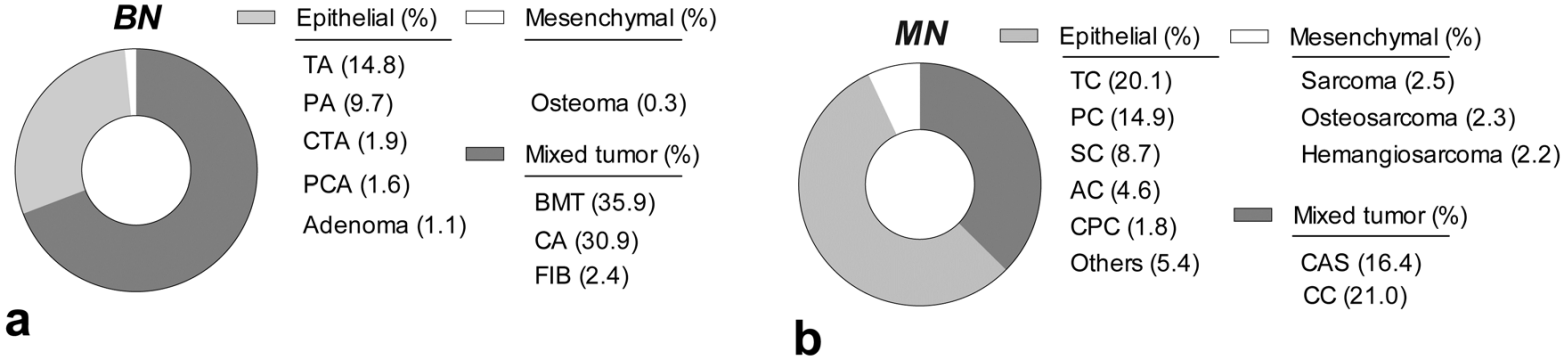
Relative frequency of the main morphological types of canine mammary tumors classified according to the tissue of origin: (a) benign and (b) malignant neoplasms.

**Fig 8:**
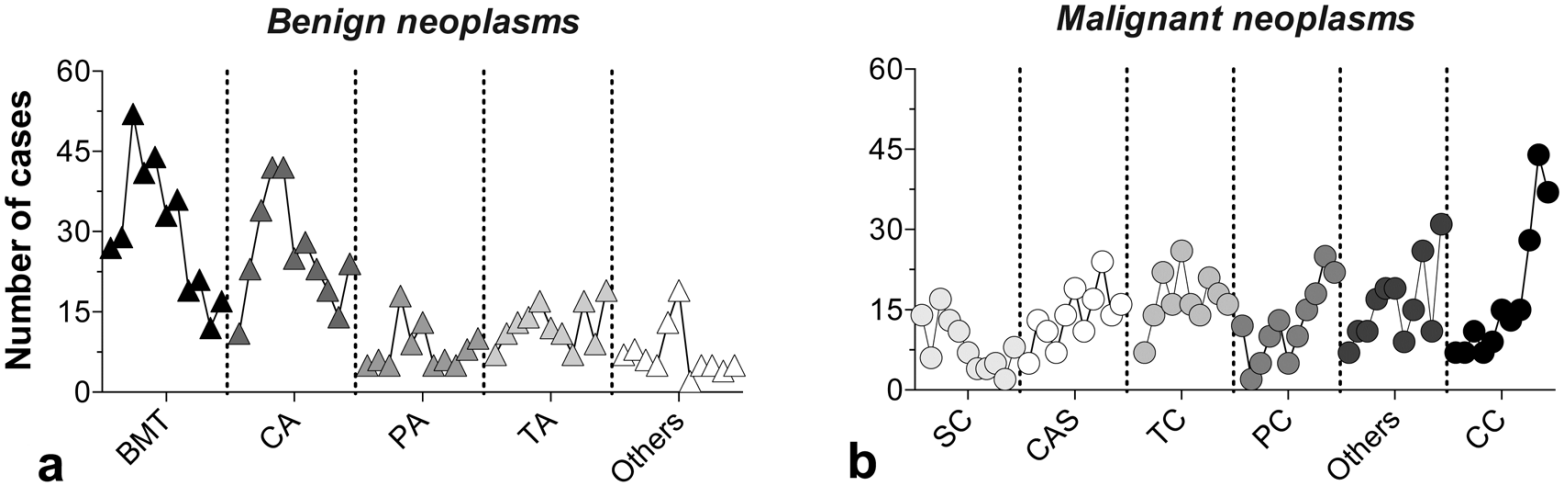
Temporal changes in the frequency of the main morphological types: (a) benign and (b) malignant canine mammary tumors.

**Fig 9:**
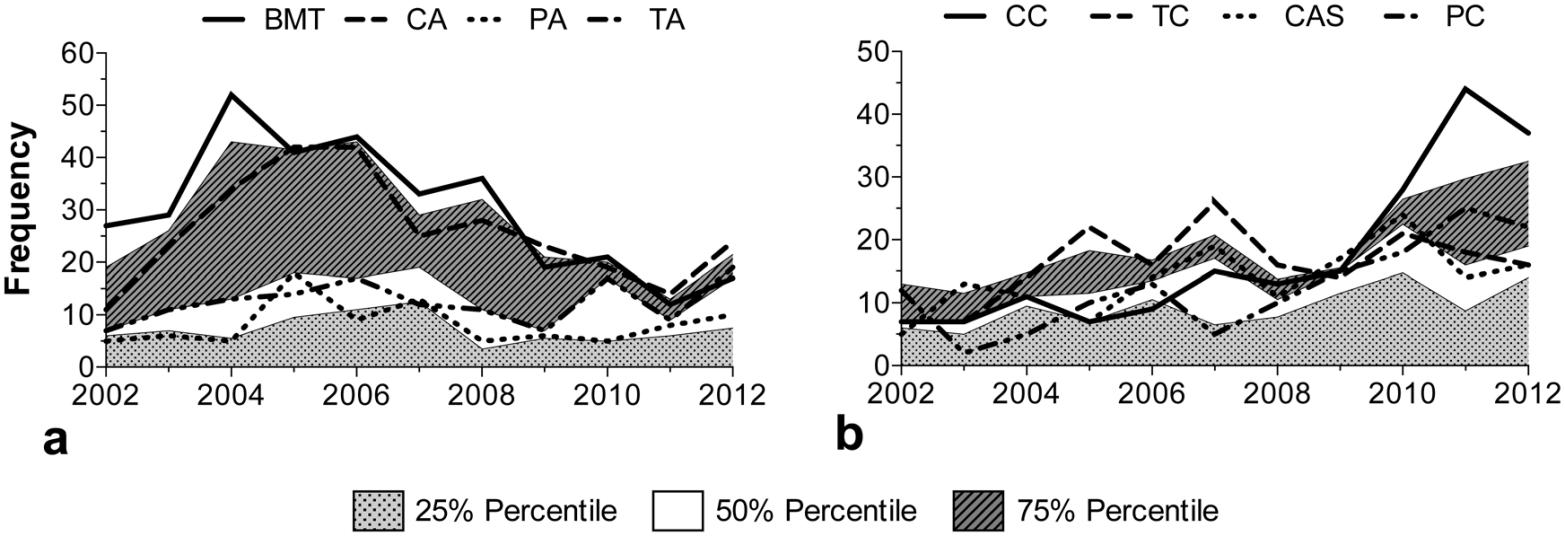
Times series showing the annual frequency allocation of the main morphological types: (a) benign and (b) MN in the epidemiological curves, formed with the 25^th^, 50^th^ and 75^th^ percentiles for the total frequency of mammary lesions.

Figs 7–9 show the relative occurrence of a variety of morphological types of CMTs. As mentioned above, mixed BN accounted for the highest number of the total cases, of which 35.9%, 30.9% and 2.4% were benign mixed tumors (BMT), complex adenoma (CA) and fibroadenoma (FIB), respectively. The most frequent benign lesions (epithelial origin) were tubular adenomas (TA), followed by papillary adenomas (PA), cystic tubular adenomas (CTA), papillary cystic adenomas (PCA) and adenoma. Hemangioma and osteoma represented the benign mesenchymal tumors (Fig 7a). From the malignant epithelial tumors group, the most frequent morphological diagnoses corresponded to tubular carcinoma (TC), papillary carcinoma (PC), solid carcinoma (SC), anaplastic carcinoma (AC) and cystic papillary carcinoma (CPC), among others.

The mixed-origin MN were complex carcinoma (CC) and carcinosarcoma (CAS). Finally, osteosarcoma, sarcoma and hemangiosarcoma were diagnosed within the mesenchymal MN (Fig 7b).

Fig 8a and 8b present the time series in relation to the BN and MN main morphological types. BNA clear reduction in BN was observed over time, and reductions also occurred for BMT and CA, representing the two main types of BN. Over time, the behavior of epithelial tumors was essentially alike. In regards to MN, four out of the five major morphological diagnoses showed sustained increases in the frequency of occurrence. Moreover, to corroborate the abovementioned epidemiological trends, the 25^th^, 50^th^ and 75^th^ percentiles of all cases of CMT for BN and MN were plotted. The information is summarized in Fig 9a and 9b. For BN, the BMT and CA curves were located above the region corresponding to the 75^th^ percentile, while the PA and TA curves were located within the 50^th^ percentile. This area comprised the average frequency of all of the types of BN (Fig 9a). It is important to note that the MN curves had higher variability due to the recent upsurge in the occurrence of this type of lesion. Indeed, the curves corresponding to CAS, CC and TC were positioned over the 75^th^ percentile, indicating an increase in the occurrence of these morphological types (Fig 9b).

### Female dog’s breed and age and their connection with the presence of canine mammary tumors

**Fig 10:**
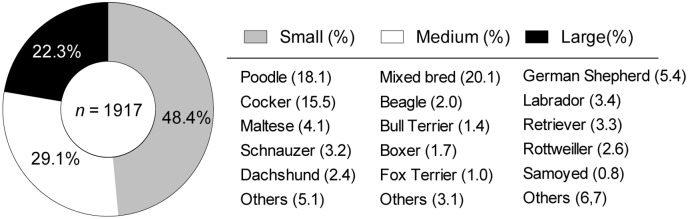
Percentage of female dogs showing mammary tumors classified according to their breed and categorized by size group.

**Fig 11:**
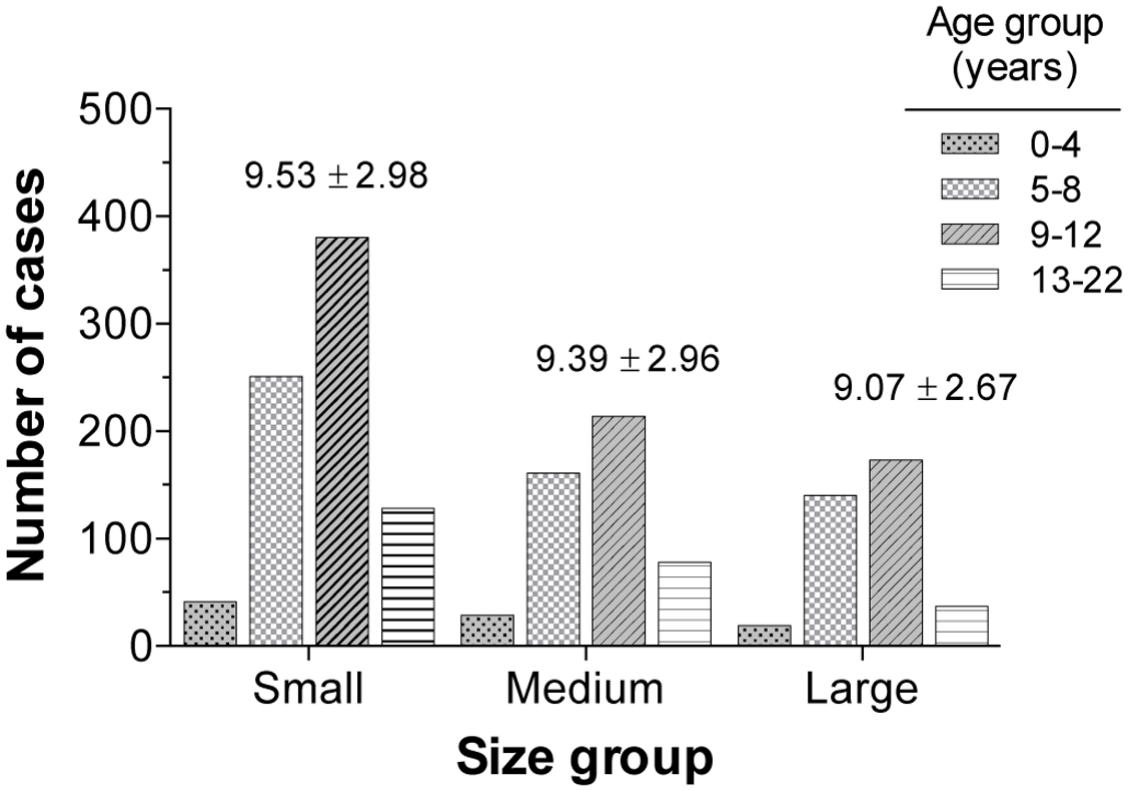
Age group distribution. Female dogs diagnosed with mammary lesions classified according to their size.

**Fig 12:**
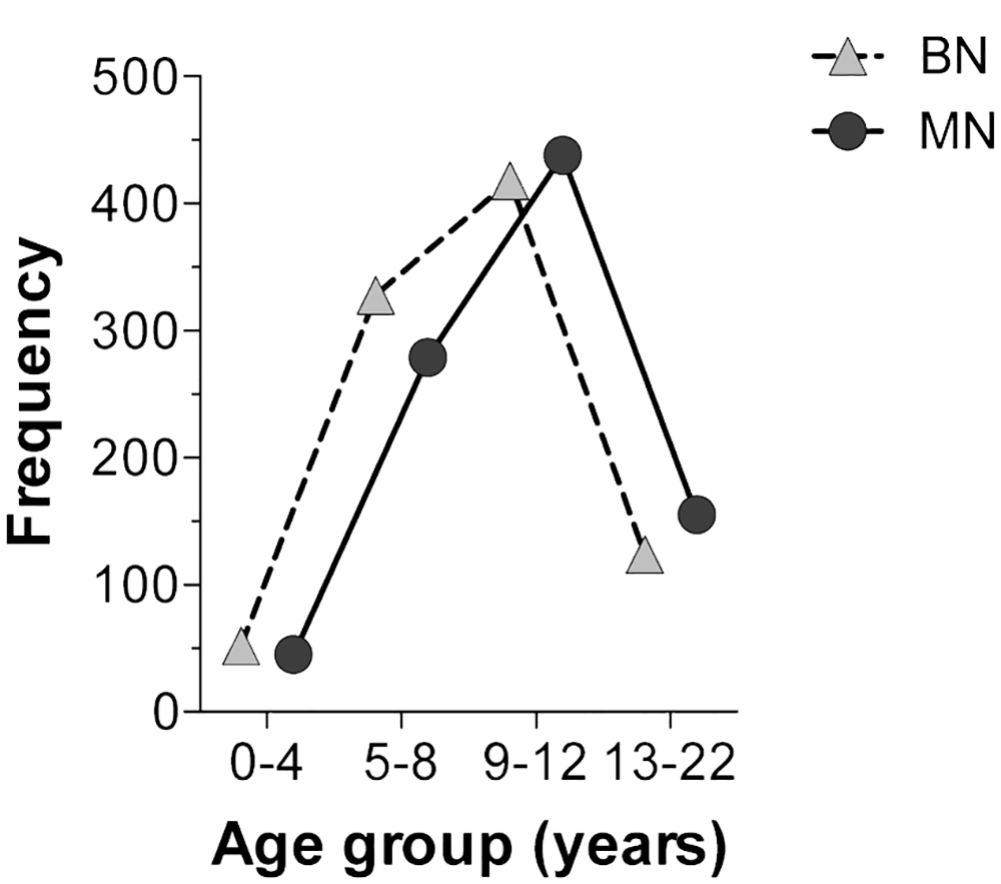
Age group distribution. Female dogs diagnosed with benign or malignant neoplasm.

Fig 10 shows a summary of the affected breeds according to animal size. The predominant category corresponded to small breed female dogs (48.4%). From this category, the Poodle and Cocker Spaniel breeds stood out in terms of frequency (n = 299 and 256, respectively), representing a total of 33.3%. Medium breeds represented 29.1% and were characterized by mixed-breed animals. This category presented the highest frequency among the total CMT; 332 cases and 20.1% of the evaluated tumors. It is important to highlight that the CMT frequency according to the breed showed a higher percentage among those phenotypically purebred animals in comparison to mixed-breed animals (80% *vs*. 20%, respectively). Finally, large-sized female dogs had the lowest frequency of occurrence (22.3%). The principal breeds within this group were the German Shepherd, Labrador Retriever and Rottweiler. No association was found (*p* > 0.05) when the occurrence of BN and MN tumors was compared to the size category. The studied population was divided into four age groups (Fig 11). Interestingly, adult animals (aged 9–12 years) showed the highest CMT frequency, followed by the group of 5- to 8-year-olds. Both groups included more than 70% of the cases. Despite obtaining these results, no significant strength of association (*p* > 0.05) between age groups and MN or BN presence (within the three evaluated sizes) was detected through *X* ^2^ analysis and the odds ratio calculation. Fig 12 shows the CMT frequency distribution according to the biological behavior for each age group. A lack of association (*p* > 0.05) between these variables is shown, as the curves registered a very similar behavior. Jointly, these results suggested that the presence of benign or MN was independent from the affected animal age and/or size.

Correspondence analysis (COA) was used to assess the strength of association between breeds, tissue of origin and morphological type of CMT. This multivariate analysis was performed according to the tumor size category and biological behavior. In this respect, COA is an extremely useful multivariate statistical tool to use to find patterns of association between nominal variables, such as those included in this work.

For small-sized breeds, the Poodle and Schnauzer showed the strongest CC and TC association, judging by the proximity between the variables included in the analysis and formed groups. Meanwhile, the Cocker Spaniel showed a higher correspondence or association with CAS and mesenchymal origin tumors.

**Fig 13:**
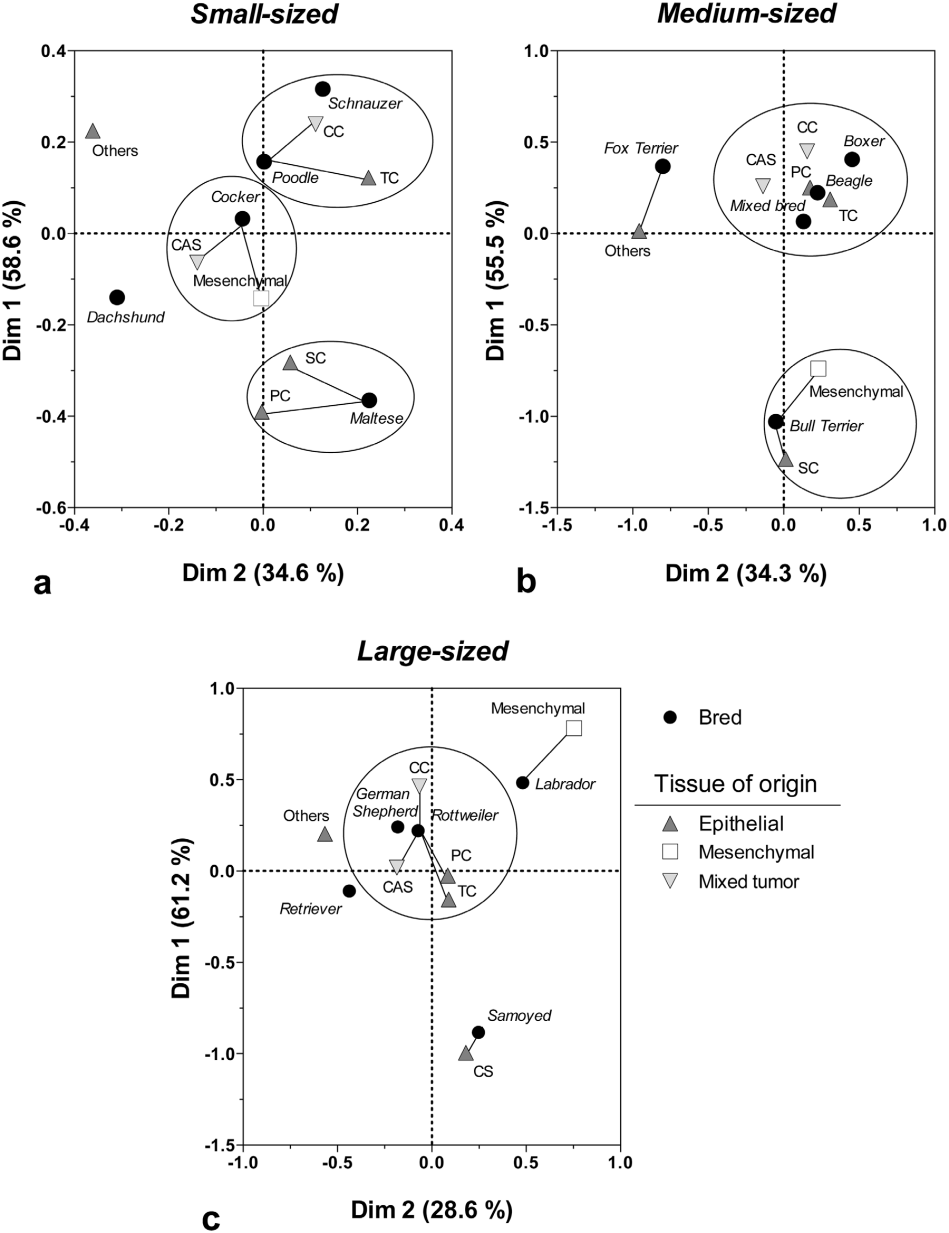
Correspondence analysis of the pattern of association between breed, morphological type and tissue of origin, in relation to canine mammary tumors: (a) small-sized, (b) medium-sized, and (c) large-sized female dogs diagnosed with malignant neoplasm. The association between variables is interpreted according to the distance that separates the adjacent categories of each variable. Thus, the lesser the distance, the greater the correspondence or association between groups of variables. Dim 1 and Dim 2 were defined as the main variance components within the multivariate analysis, accounting for 89–93% of the explanation of the total variation exhibited by the data.

Maltese dogs were mainly associated with epithelial tumors (Fig 13a). Medium-sized dogs (e.g., Mixed breed, Beagle and Boxer) formed a well-defined group, showing a strong association with mixed-origin tumors (CAS and CC) and two of the main epithelial tumors (Fig 13b).

Lastly, large-sized breeds showed a pattern consistent with the one shown in medium-sized breeds. A compact group was formed by the mixed-origin tumor and two of the main epithelial tumors (PC and CT), and these tumors were all closely associated with German Shepherds and Rottweilers (Fig 13c).

We must consider that within BN, mixed-origin tumors (MBT and CA) represent approximately 60–70% of all of the observations for all breeds. For this reason, the correspondence analysis did not show any association between breeds, tissue of origin or morphological type of cancer.

## Discussion

This study allowed us to confirm that females were mainly affected by CMT. In veterinary medicine, mammary tumors represent the most frequently diagnosed neoplasm in intact female dogs [5]. A study of the incidence of canine mammary tumors showed that tumors occurred in 0.05% of females spayed before the first heat cycle, and this incidence increased to 8% or 26% when the animals were spayed after the first or second heat. If the animal was spayed after this period of time, the risk of developing MN was the same as for an unspayed bitch [2].

It is widely known that females are predisposed to present this disease because of the tropism of natural estrogens in relation to the mammary gland that are capable of stimulating cell proliferation and generating carcinogenesis through potential cells. Likewise, it represents stimulation in transformed or pre-initiated cells in order to promote its expression [12]. Epidemiological studies performed on animals indicated that 17 beta-estradiol (E2) was linked to breast cancer. It is known that E2 can be activated by epoxidation and that it is surely capable of inhibiting DNA synthesis for DNA- binding and adduct formation (*in vivo* and *in vitro)*, playing an important role as a mammary carcinogenesis promoter [13].

CMT frequency reported in different countries may vary according to the place where the study was conducted. The study performed in Mexico (16% CMT frequency) was similar to the one conducted in Venezuela [14]. (17.1% CMT frequency). These studies both reported the presence of mammary gland tumors in 221 biopsies from dogs. Currently, in developed countries, the frequency of female dog mammary tumors has diminished due to reproductive health policies. These programs are responsible for spaying animals at an early age, and as a result, there are fewer chances of developing the disease. In female dogs, mammary cancer is a heterogeneous group in terms of morphology and biological behavior [4]. Mammary tumors represent 50% of all malignancies affecting unspayed female dogs [15]. This information is consistent with our report, which found 41–53% MN [16]. A previous study clearly showed 28 malignant and 57 BN, despite the fact that in 15 of the cases, the tumor behavior was unclassified [17].

Recently, the number of malignant diagnoses increased, and this situation can be linked to the habits of pet owners, who may expose their dogs to oncogenic substances. In recent years, the incidence of mammary tumors has increased in female dogs, perhaps due to environmental pollution and, more specifically, due to chemical exposure [18].

Furthermore, epidemiological studies showed that rodenticides were environmental pollutants involved in the development of tumors, including mammary cancer [19]. Moreover, it is extremely probable that diet, body mass and estrogens may be the cause of mammary cancer in female dogs, as in humans. When diet, fat and hormones are combined, these factors can induce or promote mammary cancer by genomic damage. The relationship between dietary fat and cancer is very complex, as certain fat metabolites binding to steroid receptors are capable of raising or lowering DNA transcription functions. Body fat deposits may also alter the hormonal balance, as adipocytes are important sources of testosterone and thus estrogen synthesis. Androgen conversion into estrogen can be proportional to the body mass and body fat degree [20].

In regards to the histological type of the CMT, this study mainly observed mixed epithelial tumors. The most common tumors were of epithelial origin, followed by mesenchymal, representing almost 1% of mammary cancers [21]. Adenocarcinoma was reported as the most common lesion (52.3%), followed by mixed tumors (44.7%) and fibrosarcoma (2.7%) [14]. Other researchers have included histologic diagnoses among seven carcinomas *in situ*: 19 simple carcinomas, 25 complex/mixed carcinomas (16 complex tubulopapillary carcinomas and four mixed types) and squamous cell carcinoma [22]. Likewise, another review was classified as single carcinoma (33.3%) and complex carcinoma (66.7%) [18]. Finally, a higher frequency of MN (78.3%)was reported as follows: simple carcinoma (28.3%), complex carcinoma (15.09%), mixed type carcinoma (15.06%) and carcinosarcoma (13.85%); second, benign:(12.27%) benign mixed tumor (4.72%), adenoma (3.3%) and fibroadenoma (2.83%); and to a lesser extent non-neoplastic lesions (8.2%) and 1.4% tumors were unclassified [23].

A number of epidemiologic studies were consistent with our report. The most affected breeds are the small breeds, according to the location of the study. Nevertheless, the problem is increasing in large-sized animals. In our study, the breeds most commonly affected by CMT were the Poodle, Cocker Spaniel, German Shepherd and Labrador Retriever, which were among the most popular breeds affected by CMT, as well as Dachshund, Yorkshire Terrier, Shih Tzu, Beagle and Boxer [24]. In other studies the most commonly reported breeds affected by CMT include Poodle, Maltese, Chihuahua, Beagle, Yorkshire Terrier, Bichon Frisé, Cocker Spaniel, English Springer Spaniel, Setter, Hound and German Shepherd [25]. A study conducted in Sweden noted that English Springer Spaniels had a clear predisposition to mammary cancer (36%), [17] followed by Spaniel Cocker, Doberman, German Shepherd and Boxer [26].

Venezuela has mainly reported CMT in purebred dogs, including Cocker Spaniel, Poodle and German Shepherd [14]. Mammary tumors were observed on 155 patients as follows: Poodles (83 cases); Cocker Spaniels (11 cases); Crossbreeds (10 cases); Boxers (6 cases); and German Shepherds (5 cases) [23]. In India, the disease was reported in crossbreed dogs (19.61%), German Shepherds (17.66%) and Labrador Retrievers (13.73%) [27].

There is a wide age range for presenting CMT. Some studies reported that CMT presented between approximately 3 and 22 years, with an average of 9 years [23]. The current study had a range from 3 to 15 years old, with an average age of occurrence from 9 to 12 years old. However, some breeds may develop CMT at younger ages. For example, the Springer Spaniel (Swedish population) had an average age of 6.9 years old [26]. Similarly, certain researchers found that in a group of 45 female dogs, the most affected were the dogs between 4 to 15 years old [22].

Another study reported the disease in dogs between 2 and 16 years old, with an average of 9 years old. The higher incidence presented in animals between 10 to 12 years old (31.37%), followed by animals between 8 and 10 years old (23.53%). A lower incidence was reported in dogs over 14 years old. The disease was not present in patients less than 2 years old [27]. In both women and female dogs, mammary tumors rarely occur before 25 years old and 5 years old, respectively [28].

Additionally, there is no statistically significant relationship between the different histologic diagnoses and breed, age, tumor size, reproductive history and exogenous hormone treatment application. This observation was congruent with our results, as we did not find a relationship between the CMT and the affected dog’s age. Nevertheless, we did observe a certain degree of association in some breeds that presented histological types of CMT. This situation requires widening the research in regards to family inherited mammary cancer through molecular genetics [23].

This study noted that the CMTs average annual frequency did not appear to have suffered any alteration (Fig 10). However, this study is important, as Mexico has manifested a canine population growth within the last few years. According to the Federation Cynologique Internationale (FCI), [10] over the past few years, the pure breeds’ records have increased. A total of 486,246 pure breed dogs were registered during 2008. In 2012, this number increased to 634,944. The INFOMEX System of Mexico City (INFOMED) stated that there are an estimated 1.2 million dogs in the city.

During April 2014, Consulta Mitofsky (a private polling firm) was in charge of conducting a survey in Mexico. This survey confirmed that over the last 3 years, a significant number of Mexican homes contained pet dogs (3%), regardless of their economic status (87.4%- low socioeconomic status and 88.6%-high socioeconomic status). The survey indicated an estimated average of 2.5 visits to the veterinary doctor per year. Owners with the lowest socioeconomic status led this practice [29]. In addition, Mexico does not have canine reproduction laws, and for this reason, Mexican people are not accustomed to neutering bitches. Often, the animal reaches the age of maturity without being spayed, becoming more susceptible to different types of diseases, including CMTs.

## Conclusions

The results of this study supported the conclusion that, in female dogs, mammary tumors are a major animal health problem that is increasing, and further development and research using veterinary oncology is required. In dogs, mammary tumors frequently present as benign and malignant lesions, often showing similar frequencies. Nevertheless, in recent years, there has been a significant tendency toward MN. It should be stressed that the most common mammary cancers were epithelial, that the most affected patients are adult females to elderly individuals, and that there was no direct relationship between age and biological behavior. Likewise, purebred small-sized (Poodle and Cocker Spaniel) female dogs had higher frequencies of CMT.
